# Research on damping performance and strength of the composite laminate

**DOI:** 10.1038/s41598-021-97933-w

**Published:** 2021-09-14

**Authors:** Bao Zhang, Zhi Li, Huawei Wu, Jinquan Nie

**Affiliations:** 1 Naveco Automobile Co., Ltd., Nanjing, 211806 China; 2 School of Automotive and Traffic Engineering, Hubei University of Arts and Science, Xiangyang, 441053 China

**Keywords:** Engineering, Materials science

## Abstract

In this paper, the influence of E-glass fiber volume fraction and laying angle on the damping and strength of composite laminates was comprehensively analyzed. By increasing the fiber laying angle and reducing the glass fiber volume fraction, the damping capacity of the composite laminate was increased, but the tensile strength of the laminate was reduced; By reducing the fiber laying angle and increasing the glass fiber volume fraction, the tensile strength of the composite laminate was increased, but the damping characteristics of the laminate was reduced. In addition, in the damping experiment of composite laminates, in order to avoid the influence of external damping sources, the vacuum non-contact damping test method was adopted in this paper, and the influence of air damping on the damping experiment results of composite laminates was comparatively analyzed. The results of comparative experiments showed that air damping had a very obvious influence on the damping of composite laminates, especially when the damping of composite laminates was small, the influence of air damping would be greater.

## Introduction

Glass fiber reinforced composite materials have the characteristics of high tensile strength, large elastic coefficient, good rigidity, and large impact energy absorption. They have been widely used in the automotive industry, machinery manufacturing, construction engineering, energy industry, aerospace, petrochemical and other fields. Damping performance is an important indicator of the dynamic performance of E-glass fiber/polyurethane composite and its structure. It can not only effectively control the vibration and noise of the mechanical structure, but also prolong the service life of the components under alternating loads. Compared with general metal materials, composite materials have a higher natural frequency, and their structure is not easy to resonate. At the same time, the matrix and fiber interface of composite materials have a greater ability to absorb vibration energy, and the effect of vibration and noise reduction is obvious. Therefore, E-glass fiber/polyurethane composite material has a broad application space in the field of vibration and noise reduction^1–4^.

As one of the effective methods to reduce vibration and noise, glass fiber reinforced composite materials have received extensive attention in recent years. Literature^5^ introduced the vibration damping model of fiber-reinforced ceramic matrix composites, and analyzed the effect of temperature on the damping performance of carbon fiber composites. Literature^6^ established a fiber-reinforced composite sheet damping model, and introduced the influence of the viscoelastic effect of epoxy resin on the damping performance. Literature^7^ introduced the influence of the stacking sequence on the mechanical properties and dynamic mechanical properties of composite materials. Literature^8^ analyzed the influence of fiber laying angle on the mechanical properties of composite materials. Literature^9^ studied the effects of reinforcement types, fiber/matrix weight ratio, and fiber laying angle on the damping performance of composite materials. Literature^10^ introduced the use of vacuum infusion molding process to prepare composite laminates, and compared the mechanical properties of ± 60° and ± 45° laminated fiber laminates. Literature^11^ discussed the effects of different layering methods and chemical treatments on the mechanical properties and free vibration properties of composite materials. However, there are few documents that consider the influence of fiber volume fraction and laying angle on the damping and strength of composite laminates at the same time. Due to the anisotropy and nonlinear characteristics of glass fiber reinforced composite materials^12^, the theoretical research on the damping characteristics of composite materials becomes more difficult, so the damping experiment becomes very important. Literature^13^ used the mechanical impedance method to study the damping characteristics of carbon fiber reinforced composite laminates. Literature^14^ tested the damping performance of composite materials through free vibration tests. Literature^15^ conducted a free vibration test using the shock frequency response test under free boundary conditions, and obtained the damping characteristics of the composite laminate. These traditional damping experiment methods cannot avoid the influence of external damping sources, such as the mass effect of the measuring sensor, air damping, friction, etc., which makes the composite material damping experiment results deviate from the actual damping.

Based on the current research status of E-glass fiber/polyurethane composite damping, the influence of the volume fraction of E-glass fiber and the laying angle on the damping and strength of the laminate was comprehensively considered in this paper. In order to improve the accuracy of the damping experiment, a vacuum non-contact damping test equipment was designed in this paper to conduct non-contact damping tests in a vacuum environment and analyze the influence of air damping on the damping test accuracy of composite materials.

## Design of the composite laminate

The design of composite material components is divided into single-layer design, laminate design and structural design. The single-layer design includes the selection of reinforcement materials and matrix, as well as the matching of their respective volume fractions. Laminate design, also known as layer design, is based on the performance of the single layer to determine the single-layer laying angle, laying order and the number of single layers in the laminated board. The structural design is to analyze and calculate the mechanical properties of the components according to the mechanical properties of the laminates to determine the geometric parameters of the components^16^. E-glass fiber/polyurethane composite laminates have been widely used in the field of vibration and noise reduction. The key parameter that affects the vibration and noise reduction effect of composite laminates is the damping coefficient of the structure. The main function of the reinforcement phase is to provide the stiffness and strength of the composite material, and to a large extent control its mechanical properties; The interface phase has a higher ability to absorb vibration, so that the composite material has a higher damping^17,18^.

The composite material is composed of a matrix phase, a reinforcement phase and an interface phase. In this paper, MAX2 polyurethane is selected as the matrix material. This type of matrix resin has high strength, tear resistance, abrasion resistance and other characteristics, and can form excellent interface bonding with glass fibers. The tensile modulus is 2800 MPa, the tensile strength is 80 MPa, the elastic elongation is 5–10%, and the critical stress intensity factor is 1.2 MPa/m^2^.

E-glass fiber is selected as the reinforcing phase of the composite laminate, which has excellent mechanical properties and can form an excellent interface with the resin matrix to improve the mechanical properties of the composite laminate. The selected E-glass fiber cloth has a density of 2.61 g/cm^3^, a tensile modulus of 81.8GPa, a tensile strength of 2570 MPa, and an elongation of 3.1%.

The performance, content and laying angle of glass fiber determine to a large extent the performance of fiber-reinforced composite materials. The glass fiber volume fraction is an important performance parameter of E-glass fiber/polyurethane laminate. If the fiber volume fraction is too small, the reinforcement effect cannot be achieved, and when the matrix strain is larger, the fiber will break. If the fiber volume fraction is too high, the strength of the composite material will be higher, but the fluidity of the resin will deteriorate, and the damping performance of the composite material will also decrease^19^. Comprehensively considering the mechanical properties of composite materials, the volume fraction of E-glass fiber is 50%, 55% and 60% respectively to synthesize composite materials.

**Figure 1:**
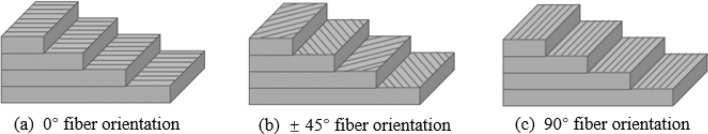
Three kinds of laying schemes.

According to the distribution of fibers in the matrix, composite laminates can be divided into unidirectional laminates and multidirectional laminates. Unidirectional laminates are fibers that are laid flat and straight in the same direction from multiple unidirectional plies; Multi-directional laminates are fibers composed of multiple unidirectional plies laid in different directions^20^. The mechanical properties of composite laminates are very complicated. In order to reduce the difficulty of laminate ply design, laying angles of 0°, ± 45°, and 90° are often used, as shown in Fig. 1.

In this paper, a vacuum infusion molding process is used to prepare E glass fiber/polyurethane laminates, and the process includes: (1) Use DHG-92 electric heating constant temperature blast drying oven to remove water from 200 × 200 mm glass fiber checkered cloth and waxed flat mold; (2) Lay the glass fiber checkered cloth in the mold cavity according to the laying angle, and then inject the prepared polyurethane glue into the sealed mold cavity. The resin completes the infiltration of the reinforcing material preform during the flow process; (3) Let it stand at room temperature, and after curing is completed, put it in an electric heating constant temperature blast drying oven at 80 °C for 3 h to cure, and demold to obtain a composite laminate.

The volume fraction of E glass fiber is 50%, 55% and 60% respectively, and the laying angle of glass fiber checkered cloth is 0°, ± 45° and 90°, respectively. The glass fiber volume fraction and the glass fiber checkered cloth laying angle were combined in pairs to prepare 9 sets of composite material laminate samples, as shown in Table 1.

**Table 1:** Samples of composite laminates.

Specimen	Volume fraction/%	Laying angle/°	Number of layers	Single layer thickness /mm	Laminate length/mm	Laminate width/mm
1#	50%	0	4	0.8	150	30
2#	55%	0	4	0.8	150	30
3#	60%	0	4	0.8	150	30
4#	50%	± 45	4	0.8	150	30
5#	55%	± 45	4	0.8	150	30
6#	60%	± 45	4	0.8	150	30
7#	50%	90	4	0.8	150	30
8#	55%	90	4	0.8	150	30
9#	60%	90	4	0.8	150	30

The E-glass fiber volume content is a key parameter that affects the properties of composite laminates. It is necessary to verify the fiber volume content of the specimens before the performance test. Since the ignition point of polyurethane is lower than that of glass fiber, the volume content of the fiber can be determined by combustion method. The testing process includes: (1) The specimen is placed in a crucible and weighed, and then the crucible is put into a muffle furnace at 625 °C. (2) The crucible is taken out and the polyurethane dust is removed after three hours. (3) The remaining glass fiber in the crucible is weighed after cooling. (4) According to the density of polyurethane and glass fiber, the fiber volume content of the specimen can be calculated. The test results show that the samples volume content meet the design requirements.

## Experimental methods

**Figure 2:**
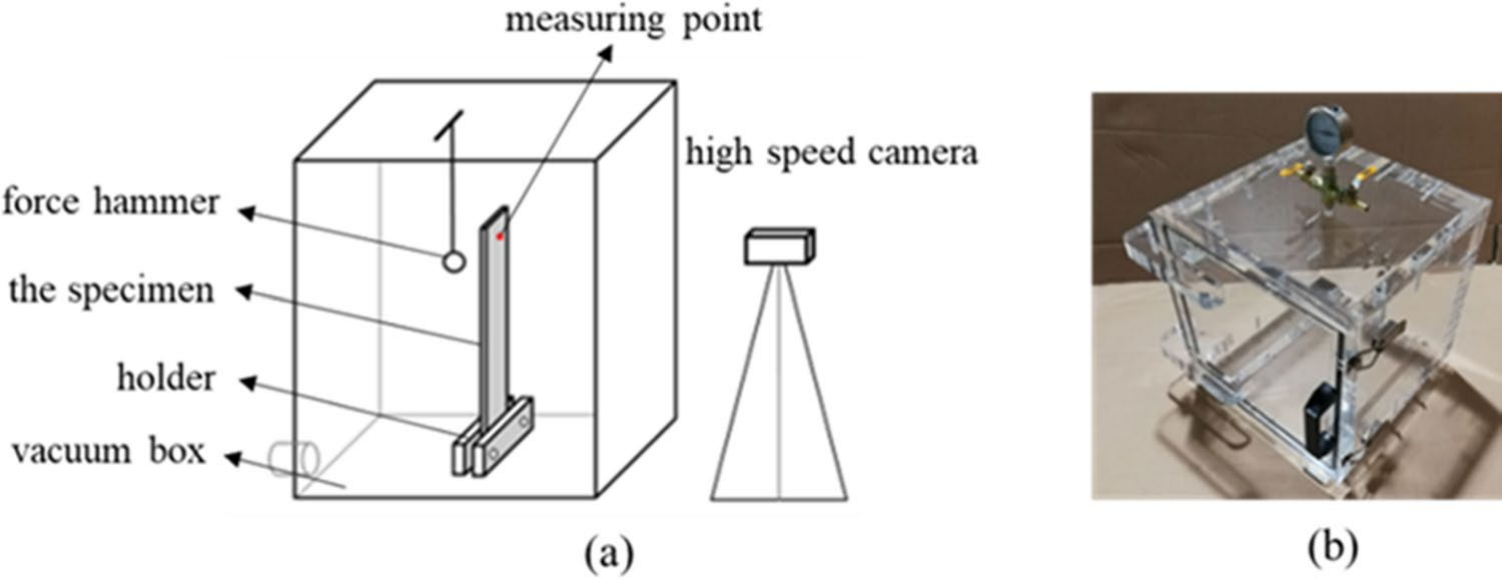
The specimen damping test system. (**a**) Glass vacuum box—schematic diagram of vacuum non-contact damping test equipment. (**b**) High-speed camera acquisition systemspeed—physical drawing of vacuum box.

The damping of composite materials is mainly due to the phenomenon of vibration energy loss caused by the mutual friction of crystal grains within the material, and its magnitude is expressed by damping coefficient or loss factor^21^. Composite material damping experiment methods mainly include torsion pendulum method, coated beam method, resonance dwell method and hammering method, etc. The damping of the material is calculated according to the vibration response of the test sample. However, composite materials have the characteristics of high stiffness, low density and nonlinearity, and the test results of different experimental methods are quite different. According to the coated beam method^22^, the composite material is pasted on the metal plate for damping test, only relatively single vibration attenuation data can be obtained, and the relationship between damping and frequency cannot be analyzed. According to the principle of resonance residence method^23^, the damping experiment of composite material samples is carried out. The test accuracy is higher than that of the Geiger plate test method, but the influence of air damping cannot be analyzed. According to the hammer method^24^, the composite material damping test can be obtained damping time-domain data and frequency-domain data, but it is impossible to analyze the influence of the sensor mass effect on the test accuracy. According to the characteristics of E-glass fiber reinforced composite material, and in order to avoid the influence of air damping and sensor mass effect on the test results, the vacuum non-contact damping test method is designed in this paper. The schematic diagram of the test equipment structure is shown in Fig. 2. The free vibration attenuation method of a cantilever beam fixed at one end is used to measure the damping coefficient of the composite laminate.

The process of measuring damping using the vacuum non-contact method includes: (1) Fix the composite material laminate in the glass box with a clamp, and mark the spot measurement point on the upper end of the laminate; (2) Use an air extractor to extract the air in the glass box, and do a good job of sealing it; (3) Adjust the movable bracket and aim the camera lens at the spot measurement point on the laminate; (4) The motor is used to control the small steel ball to hit the laminate, and the excitation point was at the upper end of the sample, so that the laminate produces free attenuation vibration, and the high-speed imaging test system is used to collect the displacement–time curve of the measurement point.

**Figure 3:**
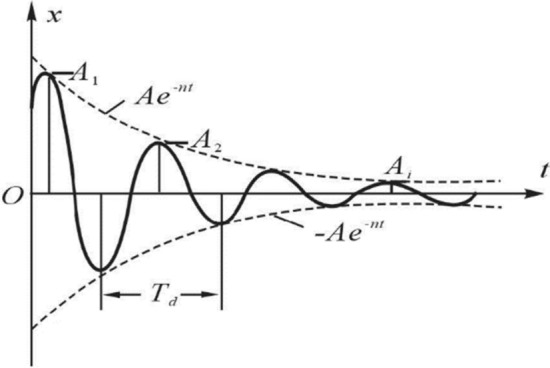
Schematic diagram of free decay curve.

The damping of a single degree of freedom system can be solved according to the amplitude attenuation ratio on the vibration attenuation curve. Figure 3 shows the free attenuation curve of a single degree of freedom system^25,26^. According to the maximum displacement A_i_ sampled in each period and the maximum displacement A_i+1_ of the next adjacent period, the vibration logarithmic attenuation rate δ is calculated according to formula (1), and the corresponding damping coefficient ξ is calculated according to formula (2).

1 $$\updelta =\mathrm{ln}\frac{{A}_{i}}{{A}_{i+1}}=\frac{2\pi \xi }{\sqrt{1-{\xi }^{2}}}$$

2 $$\upxi =\frac{\delta /2\pi }{{\left(1+\delta /2\pi \right)}^{2}}$$

When the system damping is large, the adjacent amplitude changes on the vibration attenuation curve will be obvious. The amplitude data of the first cycle and the second cycle can be used to accurately calculate the damping of the system. When the system damping is small, the adjacent amplitude changes on the vibration attenuation curve will be small, which will bring inconvenience to the processing of measurement data. In order to improve the calculation accuracy of system damping, the vibration logarithmic attenuation rate δ can be calculated according to (3) according to the amplitude of the i period amplitude A_i_ and the i + n period amplitude A_i+n_, and then perform arithmetic average processing on all logarithmic decay rates, and then calculate the system damping ratio according to formula (2).

3 $$\updelta =\frac{1}{n}\mathrm{ln}\frac{{A}_{i}}{{A}_{i+n}}$$

## Results and discussion

### Analysis of the influence of E-glass fiber volume content on damping

**Figure 4:**
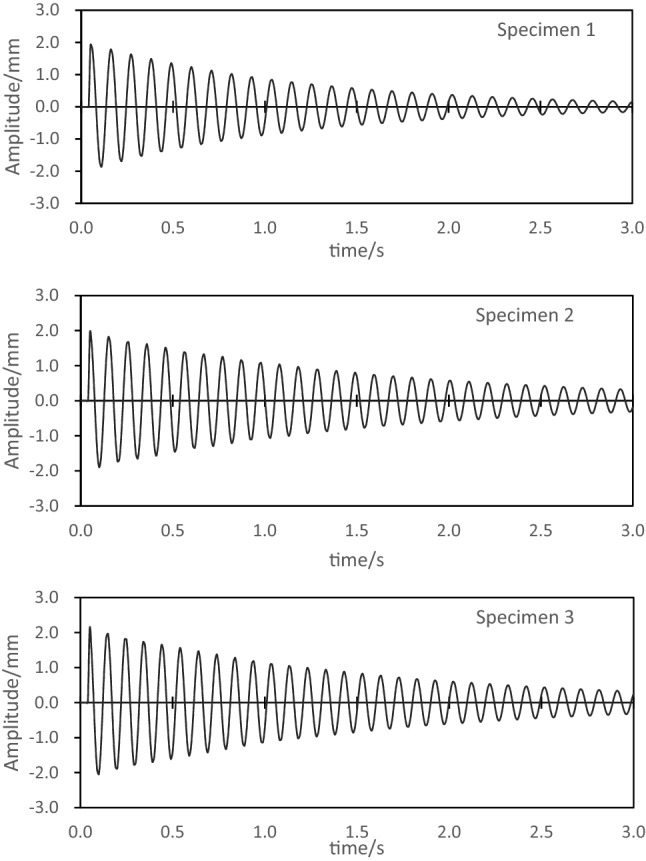
Damping experiment of samples with different fiber volume fraction.

The main contribution of E-glass fiber/polyurethane laminate damping comes from the polyurethane matrix, but E-glass fiber is a polymer material, and the material itself has a relatively high damping. Therefore, it is necessary to consider the influence of E-glass fiber on the damping of composite laminates. In this section, specimens 1, 2, and 3 with a fiber laying angle of 0° are selected for comparative experiments of composite laminate damping, and the free vibration attenuation curves of the three specimens are shown as Fig. 4. According to formula (3), the damping coefficient of specimen 1 is calculated to be 1.45%, the damping coefficient of specimen 2 is 1.36%, and the damping coefficient of specimen 3 is 1.15%. When the volume fraction of E-glass fiber increases from 50 to 55%, the damping coefficient of the specimen decreases by 6.2%. When the volume fraction of E-glass fiber increases from 50 to 60%, the damping coefficient of the specimen decreases by 20.7%. The test results show that the E-glass fiber volume fraction has an effect on the damping of the composite laminate, and there is a nonlinear relationship between the damping coefficient of the specimen and the E-glass fiber volume fraction. When the laying angle is the same, the damping coefficient of the specimen with the lower E-glass fiber volume fraction is large, and the damping coefficient of the specimen with the higher volume fraction is small. The main reason for the above phenomenon is that when the volume fraction of E-glass fiber is high, the polyurethane content will decrease, and the viscoelasticity of the composite material will subsequently deteriorate. Therefore, composite laminates with lower E-glass fiber volume fraction have better vibration damping performance.

### Analysis of the influence of E glass fiber ply angle on damping

**Figure 5:**
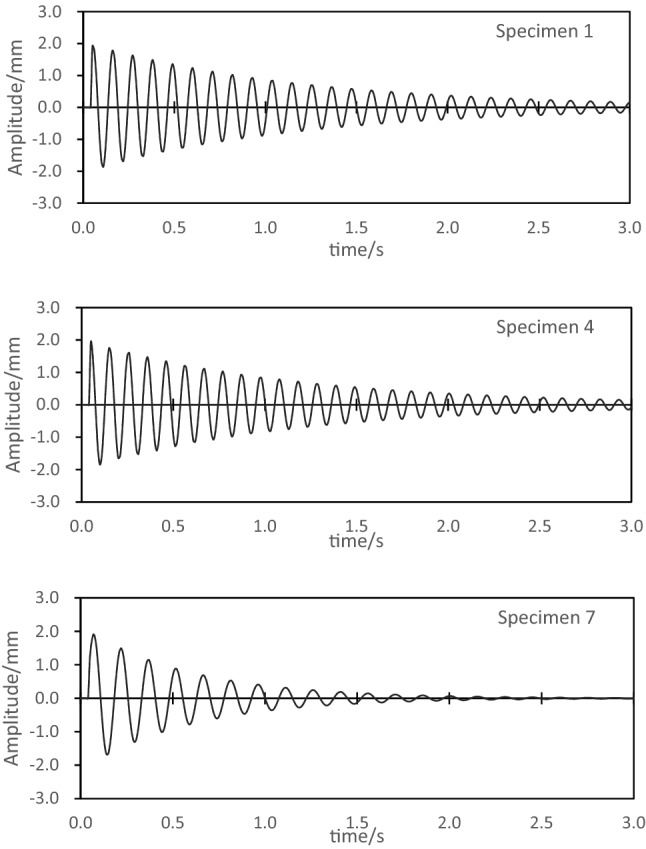
Damping experiment of samples with different laying angles.

The angle of the E-glass fiber layup will affect the overall stiffness of the laminate. When the stiffness is too high, it will affect the deformation ability of the polyurethane matrix, hinder the relative sliding between the reinforcement and the interface, and reduce the damping characteristics of the laminate^27^. In this section, specimens 1, 4, and 7 with a fiber volume fraction of 50% are selected for comparative experiments of composite laminate damping, and the free vibration attenuation curves of the three specimens are shown as Fig. 5. The damping coefficient of specimen 1 is 1.45%, the damping coefficient of specimen 4 is 1.73%, and the damping coefficient of specimen 7 is 2.42%. The damping coefficient of specimen 4 is 19.3% higher than that of specimen 1, the damping coefficient of specimen 7 is 66.9% higher than that of specimen 1. The test results show that the effect pf laying angle on the damping coefficient is very obvious, especially in the range of 45°–90°, and the increase of the laying angle can significantly improve the damping coefficient of the composite laminate.

Specimen 1 adopts a 0° laying method. The fiber layer plays a major role in resisting external forces. The laminate structure has a large rigidity. Under the same excitation conditions, the composite material has less internal friction dissipation and a small damping coefficient. Specimen 7 adopts a 90° laying method. The polyurethane layer plays a major role in resisting external forces, resulting in low rigidity of the laminate structure. Under the same excitation conditions, the composite material has more internal friction dissipation and a large damping coefficient. Specimen 4 adopts ± 45° alternate laying method, and its structural rigidity is between 0° and 90° laying method.

### Analysis of the influence of air damping on the damping of composite laminates

**Figure 6:**
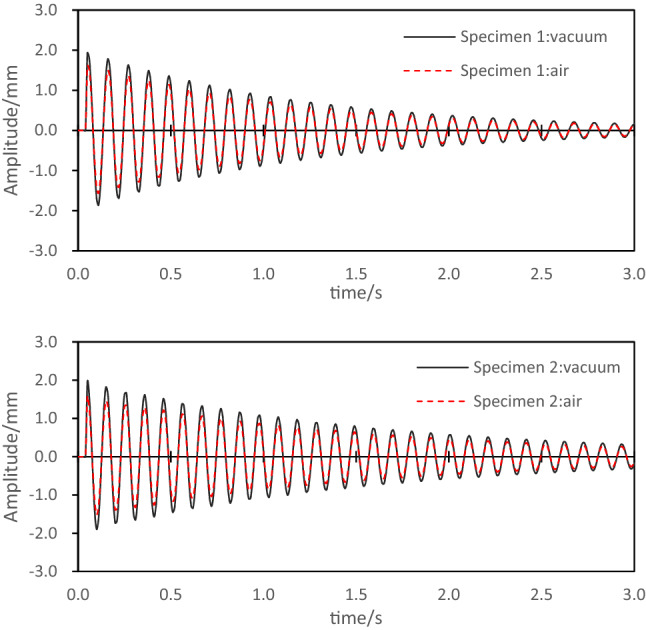
Damping test of consider air.

Because composite materials have the characteristics of low density and relatively small damping, if the damping experiment of composite materials is carried out in a natural environment, then the influence of air damping on the experiment cannot be ignored, especially when the low-frequency and high-amplitude excitation is performed, the impact of the air damping will be more obvious^28,29^. In this section, specimens 1 and 2 with a laying angle of 0° are selected for damping tests in air and vacuum respectively, and the free vibration attenuation curves of the two specimens are shown as Fig. 6. The test results show that air damping will accelerate the vibration attenuation of the specimen. The damping coefficient of specimen 1 in vacuum is 1.45%, and the damping coefficient in air is 1.73%. Air damping increases the damping coefficient of specimen by 19.1%. The damping coefficient of specimen 2 in vacuum is 1.36%, and the damping coefficient in air is 1.78%, and air damping increases the damping coefficient of specimen 2 by 24.7%. Therefore, in the damping experiment in the natural environment, air damping has a very obvious influence on the damping of the composite laminate, but the air damping does not change the vibration frequency of the laminate. By comparing the degree of influence of air damping on the damping coefficients of specimens 1 and 2, it can also be found that when the composite laminate has a smaller self-damping, the air damping will have a greater influence on the experimental results.

### Strength analysis of composite laminates

**Figure 7:**
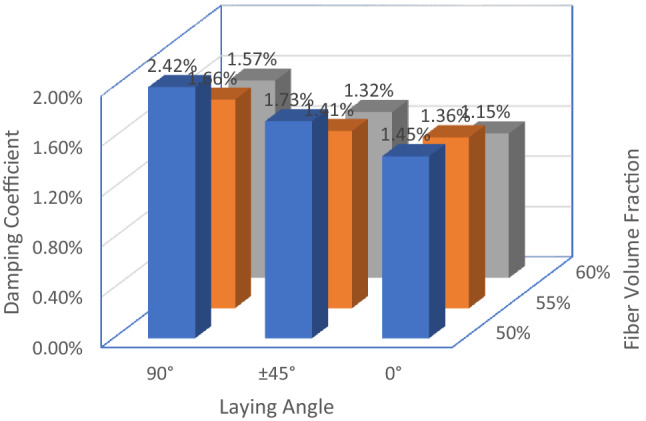
Comparison chart of damping coefficient.

The comparison results of the damping coefficients of the 9 groups of specimens are shown as Fig. 7. Data analysis shows that the volume fraction of E-glass fiber has a greater impact on the damping of the laminate, and the larger the volume fraction, the smaller the damping coefficient. E-glass fiber laying angle is in the range of 45°-90°, and increasing the laying angle can significantly improve the damping coefficient of the laminate. The maximum damping coefficient of specimen 7 is 2.42%; the minimum damping coefficient of specimen 3 is 1.15%.

**Figure 8:**
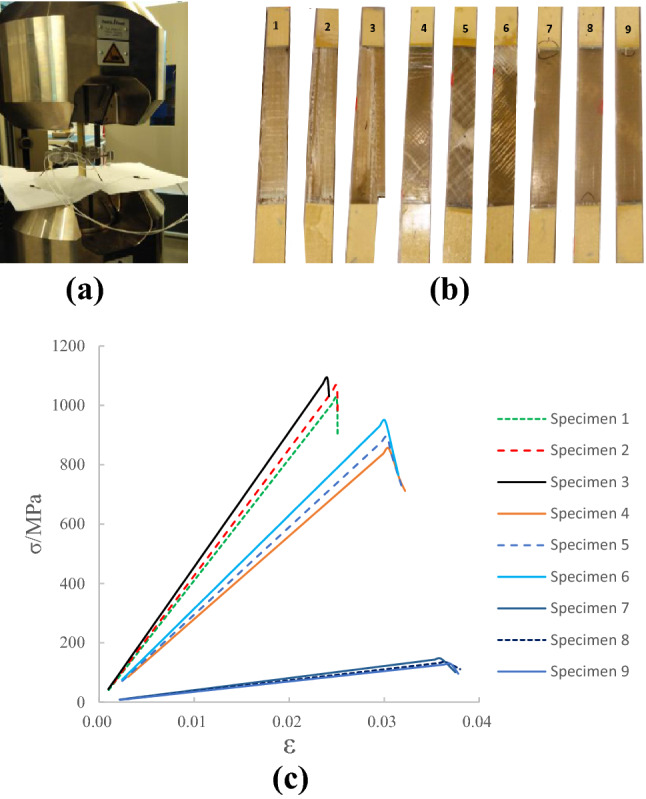
Strength test of the specimens. (**a**) Tensile test equipment. (**b**) Tensile failure photos of specimens. (**c**) Quasi-static tensile stress–strain curve.

In the damping design of composite laminates, structural strength should be considered simultaneously, and structural strength should not be sacrificed in exchange for structural damping. Therefore, it is necessary to conduct tensile strength experiments on 9 sets of specimens to analyze the influence of the change in the volume fraction of E glass fiber and the laying angle on the structural strength of the laminate. In this paper, the two-way Clip-on extensometer is used to test the tensile strength of the specimen, as shown in Fig. 8a. Figure 8b is a photo of tensile failure test of E-glass fiber/polyurethane laminate test piece. During the stretching process of specimens 1, 2, and 3, longitudinal cracks first appeared in the relatively affluent areas of polyurethane, and the longitudinal ends of the cracks expanded, causing tearing failure between glass fibers and irregular cross-sections, and most of the sections were serrated. The initial cracks of specimens 4, 5, and 6 are alternating positive and negative, and the cracks extend in the direction of ± 45°, and the glass fiber fails to pull off from the matrix, and the cross section is in a dovetail shape. Specimen 7, 8, and 9 will have transverse cracks during the stretching process, and the cracks will expand on both sides of the longitudinal direction, which will be broken near the reinforcing sheet, and the section will be relatively flat.

The stress–strain level of E-glass fiber/polyurethane laminate is between the stress and strain of E-glass fiber and polyurethane, and its value is related to the structure size of the laminate, the volume fraction of E-glass fiber and the laying angle^30^. The stress–strain curves of specimens are shown as Fig. 8c, and the curves show that the stress and strain of specimens are linear elastic before failure. The tensile failure of specimens is instantaneous fracture failure. E-glass fiber laying angle has a very obvious influence on the tensile stress of the laminate. Under the same fiber volume fraction, the maximum tensile stress that the specimens can withstand decreases as the laying angle becomes larger. This is because when the 0°-laminated specimen is subjected to a tensile load, since the direction of the tensile force is parallel to the direction of the glass fiber, the number of fibers participating in the load-bearing is the largest, and the tensile strength is the largest. The direction of the glass fiber of the 90° laid specimen is perpendicular to the direction of tensile force, which is mainly carried by the polyurethane matrix, and the tensile strength is the weakest. The load-bearing capacity of ± 45° alternately laid specimens is between 0° and 90° laid specimens.

The curves in Fig. 8c also show that the specimens are alternately laminated at 0° and ± 45°, and increasing the fiber volume fraction will enhance the tensile strength of the specimens, and this is because when the E-glass fiber volume fraction increases, the number of glass fibers participating in the load will increase, and the tensile strength will increase. However, the increase of fiber volume fraction will reduce the tensile strength of the 90° laid specimen, and this is because the 90° laid specimens are mainly carried by the matrix, and when the fiber volume fraction increases, the polyurethane matrix content will decrease, and the tensile strength will decrease.

The maximum tensile strength of specimen 3 is 1093.4 MPa, and the minimum tensile strength of specimen 9 is 129.1 MPa. The tensile strength of specimen 3 is 8.5 times of the tensile strength of specimen 9. In terms of improving strength, the combination of 0° layer and high fiber volume fraction is the most advantageous; in terms of improving damping, the combination of 90° layer and low fiber volume fraction is the most advantageous; Therefore, in the damping design of E-glass fiber/polyurethane laminates, it is necessary to comprehensively consider the use environment and strength, and match the optimal combination of E-glass fiber laying angle and volume fraction.

## Conclusion

In this paper, vacuum infusion molding process was used to prepare 9 groups of specimens with E-glass fiber volume fractions of 50%, 55%, and 60%, and laying angles of 0°, ± 45°, and 90°, and the effects of fiber volume fraction and laying angle on the damping and strength of composite laminates were analyzed through experiments. The lower the fiber volume fraction, the greater the laying angle, the better the damping performance of the composite laminate, but the worse the tensile strength; The higher the fiber volume fraction, the smaller the laying angle, the worse the damping performance of the composite laminate, but the better the tensile strength; The 0° fiber layer is beneficial to improve the strength of the laminate; the 90° fiber layer is beneficial to the damping performance of the laminate; the performance of the ± 45° fiber alternate layer is between 0° and 90° layer. When performing the damping design of E-glass fiber/polyurethane laminate, it is necessary to comprehensively consider the use environment and strength, and match the optimal combination of E-glass fiber laying angle and volume fraction.

In order to improve the damping experiment accuracy of composite laminates, a vacuum non-contact damping test equipment was designed in this paper to perform non-contact damping tests in a vacuum environment, and the influence of air damping on the damping experiments of composite laminates was analyzed. Air damping speeds up the vibration attenuation of the specimen, and has a greater impact on the damping experiment results of the specimen, especially the impact on the specimen with small damping, but will not improve the vibration period of the specimen.

## Data Availability

The CHANCES participating cohorts’ data are available only to the collaborating scientists from the respective CHANCES participating centers. The data may be available upon request for some of the participating centers but not for all due to relevant data protection laws.
